# Narrative Review on Echocardiographic Evaluation of Patent Ductus Arteriosus in Preterm Infants

**DOI:** 10.3390/jcdd11070199

**Published:** 2024-06-28

**Authors:** Yogen Singh, Belinda Chan, Shahab Noori, Rangasamy Ramanathan

**Affiliations:** 1 Department of Pediatrics, Division of Neonatology, University of California—UC Davis Children’s Hospital, Sacramento, CA 95817, USA; 2 Department of Pediatrics, Division of Neonatology, University of Utah, Salt Lake City, UT 84132, USA; belinda.chan@hsc.utah.edu; 3 Division of Neonatology, Fetal and Neonatal Institute, Department of Pediatrics, Children’s Hospital Los Angeles, Keck School of Medicine, University of Southern California, Los Angeles, CA 90033, USA; snoori@chla.usc.edu; 4 Department of Pediatrics, Division of Neonatology, University of Southern California, Los Angeles, CA 90048, USA; rangasamy.ramanathan@cshs.org; 5 Department of Pediatrics, Division of Neonatology, Cedars Sinai Guerin Children’s, Cedars Sinai Medical Center, Los Angeles, CA 90048, USA

**Keywords:** patent ductus arteriosus (PDA), preterm infant, premature infants, clinical diagnosis, echocardiographic evaluation of PDA

## Abstract

Persistent Patent Ductus Arteriosus (PDA) is prevalent among extremely preterm infants, with its occurrence inversely related to gestational age. A persistent PDA correlates with increased mortality and morbidities such as intraventricular hemorrhage, pulmonary hemorrhage, chronic lung disease, bronchopulmonary dysplasia, and necrotizing enterocolitis as observed clinically. Conversely, numerous randomized controlled trials have failed to demonstrate significant benefits from PDA treatment. One contributing factor to these conflicting findings is that PDA affects each individual differently depending on the cardiovascular decompensation and its hemodynamic impact. PDA management should be based on the hemodynamic significance, rather than just the presence or size of PDA. This comprehensive narrative review paper describes echocardiographic parameters that allow a better understanding of the hemodynamic impact of PDA. A newer modality, like lung ultrasound, is also described here as an adjunct to assess the PDA impact on the lungs from pulmonary overcirculation.

## 1. Introduction

The diagnosis and management of Patent Ductus Arteriosus (PDA) varies [1]. While PDA in most preterm infants closes spontaneously soon after birth, it persists longer among extremely preterm infants, affecting approximately 60–66% of those born before 28 weeks of gestation [1,2,3]. Notably, the prevalence of PDA increases with lower gestational age [2]. Additional risk factors include low birth weight, absence of antenatal steroids, respiratory failure on mechanical ventilation, sepsis, and excessive fluid overload [4,5].

In clinical observation studies, persistent PDA is associated with high mortality and morbidities, including prolonged hospital stays, intraventricular hemorrhage (IVH), pulmonary hemorrhage, chronic lung disease (CLD), bronchopulmonary dysplasia (BPD), and necrotizing enterocolitis (NEC) [6,7]. However, numerous randomized controlled trials have not shown improvement in long-term outcomes with PDA treatment. Presently, consensus is lacking on whether, when, and how to treat PDA in preterm infants [8,9,10,11,12,13].

This paper focuses on the clinical diagnosis of PDA, echocardiographic assessment, and determination of its hemodynamic significance, especially on the echocardiographic parameters that can aid bedside clinicians in making timely and appropriate decisions. Other manuscripts in this special issue on “Patent Ductus Arteriosus in Premature Babies” address the management of PDA and its treatment options.

## 2. Clinical Diagnosis of PDA

Infants with a PDA may initially remain asymptomatic due to elevated pulmonary vascular resistance (PVR), impeding excessive blood flow between the aorta and the pulmonary artery across the PDA. The PDA becomes hemodynamically significant when blood shunting across the PDA increases, causing strain on the other organ systems. Pulmonary overcirculation occurs when PVR diminishes over subsequent days after birth, resulting in symptoms such as pulmonary edema, tachypnea, desaturations, or apnea. Increased pulmonary venous return may lead to cardiac manifestations, including a loud heart murmur, tachycardia, cardiomegaly, and a hyperactive precordium. The phenomenon of blood flow diversion from the systemic circulation, known as the “steal phenomenon,” results in bounding peripheral pulses, widened pulse pressure, lowered diastolic blood pressure, and systemic hypoperfusion, often characterized by oliguria and feeding intolerance. When myocardial adaptation to hemodynamic significant PDA (hsPDA) fails, heart failure may lead to severe hypotension and acidosis, necessitating cardioactive or vasopressor medications [12,14,15].

Not all infants with persistent PDA exhibit all the signs and symptoms. The clinical presentation depends on several factors: (1) the size of the ductus arteriosus, (2) the direction and magnitude of shunt volume across the PDA are influenced by the interplay of vascular resistance and pressure between systemic and pulmonary circulation, (3) myocardial response to increased shunt volume, and (4) end-organ adaptability to mitigate the steal phenomenon [11,12,14]. Echocardiography can better evaluate these factors, as explained later in this review (see below). Non-specific symptoms may also resemble other critical congenital heart defects (CHD), making diagnosis challenging solely based on clinical examination [16]. Therefore, echocardiographic assessment is critical for the clinical management of preterm infants and is recommended before considering any intervention.

## 3. Echocardiography for Diagnosing PDA and Assessing Hemodynamic Significance

Echocardiography remains the gold standard bedside tool for diagnosing PDA. It confirms its presence and assesses the PDA shunt volume and downstream effects. Before treating PDA, it is crucial to exclude any underlying CHDs, particularly duct-dependent defects. 

**Figure 1:**
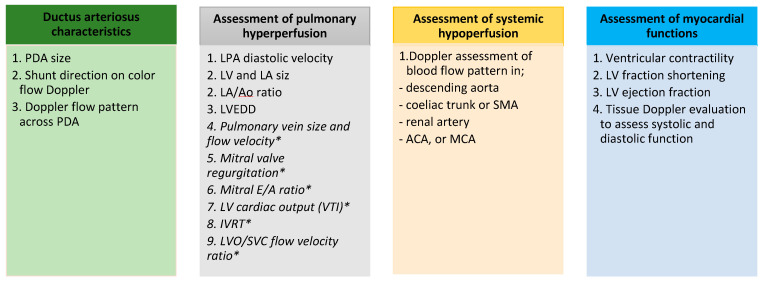
Summary of the echocardiographic assessment of PDA and its hemodynamic impacts. The parameters labeled with * are less commonly used for echocardiographic assessment. *ACA*—*anterior cerebral artery, Ao*—*aorta, Coeliac*—*coeliac trunk, E/A*—*early diastolic flow velocity (E velocity) vs. late diastolic transmitral flow velocity (A velocity), IVC*—*inferior vena cava, IVRT*—*isovolumic relaxation time, MCA*—*posterior cerebral artery, L→R, left-to-right shunt, LA*—*left atrium, LPA*—*left pulmonary artery, LV*—*left ventricle, LVEDD*—*left ventricular end-diastolic diameter, pulmonary artery LVO*—*left ventricular cardiac output, PA*—*pulmonary artery, PDA*—*patent ductus arteriosus, PV*—*pulmonary vein, RA*—*right atrium, RV*—*right ventricle SMA*—*superior mesenteric artery, SVC*—*superior vena cava, VTI*—*velocity time integral*.

**Figure 2:**
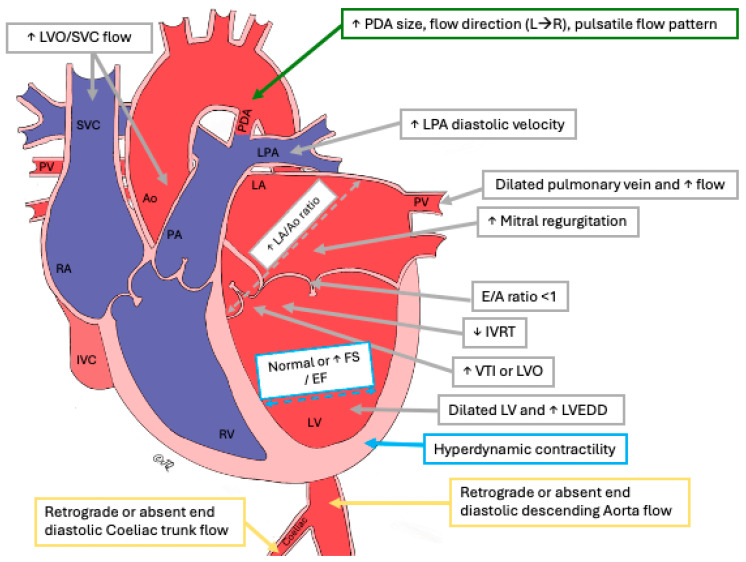
A simple diagrammatic representation of the impacts of the shunt across the patent ductus arteriosus on various end-organs. *Ao-Aorta, E/A early diastolic flow velocity (E velocity) vs. late diastolic velocity (A velocity), EF—ejection fraction, FS—fraction shortening, IVC—inferior vena cava, IVRT—isovolumic relaxation time, L→R, left to right shunt, LA—left atrium, LPA—left pulmonary artery, LV—left ventricle, LVEDD—left ventricular end-diastolic diameter, LVO—left ventricular cardiac output, PA—pulmonary artery, PDA—patent ductus arteriosus, PV—pulmonary in, RA—right atrium, RV—right ventricle, SVC—superior vena cava, VTI—velocity time index*.

A comprehensive echocardiographic assessment of PDA and its hemodynamic significance typically encompasses the following aspects: (a) ductus arteriosus characteristics, (b) assessment of pulmonary hyperperfusion, (c) assessment of systemic hypoperfusion and (d) assessment of myocardial functions (Figure 1 and Figure 2) [11,12,17].

The following sections outline the echocardiographic parameters commonly used for the comprehensive assessment of PDA. They also explain the physiological basis of these measurements and detail the methods of obtaining them.

### 3.1. Echocardiographic Assessment of Ductal Arteriosus Characteristics 

Echocardiography can determine the PDA’s size, morphology, shunt direction, and flow pattern. 

#### 3.1.1. PDA Size

**Figure 3:**
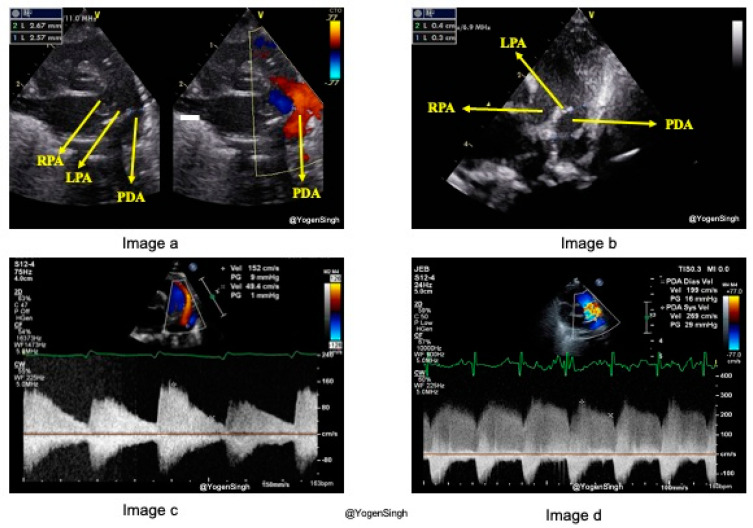
Characteristics of patent ductus arteriosus (PDA). (**a**) shows the measurement of PDA simultaneously on 2D and color flow mapping. (**b**) shows the measurement of a tortuous PDA in 2D; the narrowest part should be reported. (**c**) shows the pulsatile flow pattern on Doppler assessment, and (**d**) shows a constricting flow pattern. *LPA—left pulmonary artery, RPA—right pulmonary artery, PDA—patent ductus arteriosus*.

Describing the size of PDA as small, moderate, or large over-simplifies the echocardiographic evaluation of ductal characteristics, its morphology, and hemodynamic impact. PDA size is measured from the transductal diameter at the site of maximum constriction, most commonly seen at the pulmonary end (Figure 3a). PDA is not a straight symmetric tubular vessel, but it has six different types of morphologies and is tortuous at times (Figure 3b). PDA constriction leading to closure can occur at any segment of the ductus arteriosus, although commonly seen at the pulmonary end [17,18]. The standard echocardiography views to measure the size of a PDA are: (1) high left-sided parasternal “ductal” view, or (2) suprasternal ductal cut view. A PDA’s size can be measured on a 2D image or using Color Doppler (color flow mapping). Color Doppler can be used to highlight the PDA’s size for better visualization. However, the color gain setting should be optimized to avoid color “bleeding” out of the vessel lumen, leading to overestimating the PDA diameter. A PDA diameter of >2 mm is generally considered large in preterm infants.

#### 3.1.2. PDA Shunt Direction

The flow direction across PDA depends upon the gradient between pulmonary and systemic pressures and the interplay between systemic and pulmonary vascular resistance. By convention, blood flow from the aorta to the pulmonary artery is left-to-right when systemic pressure is higher than pulmonary pressure, and the magnitude of blood flow mostly correlates with the hsPDA. When the PVR is high or in certain CHDs (such as interrupted aortic arch), the PDA flow can be bidirectional or right-to-left. The direction of the ductal shunt is assessed using Color Doppler. In a conventional echocardiography setting, the left-to-right shunt across the PDA on a high parasternal ductal view is seen as a red jet (Figure 3a), while a right-to-left ductal flow is seen as blue in color. The Doppler assessment shows a left-to-right shunt above the baseline (toward the probe), while the right-to-left shunt is seen below the baseline (away from the probe) [17,19].

#### 3.1.3. PDA Shunt Velocity and Pattern

The clinical and hemodynamic significance of PDA depends on the dynamics of blood flow across the ductus, which is contingent upon factors such as the pressure gradient, vessel dimensions, vessel length, and blood viscosity. The Doppler assessment of flow velocity (volume per unit time) and flow patterns across the PDA offers invaluable insights. A lower pressure gradient between systemic and pulmonary circulation or a large PDA allows blood to flow at a lower velocity through the ductus, resulting in a non-restrictive or pulsatile shunt flow pattern characterized by low peak systolic velocity and a very low diastolic velocity (<50 of peak systolic velocity) (Figure 3c). Conversely, as a PDA is constricting or closing, a higher peak velocity is seen across the constricting segment, known as a restrictive or constricting flow pattern (Figure 3d) [20]. The shunt velocity varies during the cardiac cycle. If the end-diastolic velocity exceeds 50% of the peak systolic velocity, it is considered a constricting flow pattern. The flow velocity can be obtained by applying pulsed or continuous wave Doppler within the PDA. Although pulsed wave Doppler is specific (Figure 2), continuous wave Doppler has to be applied to avoid aliasing when the velocity is >2 m/s. 

### 3.2. Echocardiographic Assessment of Pulmonary Over-Circulation

**Figure 4:**
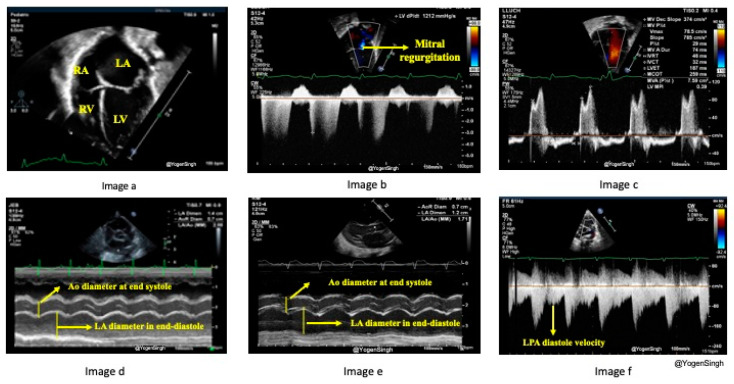
Echocardiography assessment of pulmonary over-circulation. (**a**) shows the left heart is larger than the right heart due to volume overload in the apical four-chamber view. (**b**) shows the mitral regurgitation on color flow mapping from the apical four-chamber view. (**c**) shows E/A ratio inversion across the mitral valve inflow. (**d**) shows the increased LA to Aorta (LA/Ao) ratio in the parasternal short-axis view on the M-mode. (**e**) shows the increased LA to Aorta (LA/Ao) ratio on the M-mode in the parasternal long-axis view. (**f**) shows an increased diastolic velocity in the left pulmonary artery (LPA). *LA—left atrium, LV—left ventricle, RA—right atrium, RV—right ventricle, Ao—aortic valve, LPA—left pulmonary artery*.

The increase in pulmonary blood flow caused by the PDA may not be directly measured at the bedside, but it can be estimated with surrogate echocardiographic parameters. A significant PDA shunt volume leads to turbulence in branch pulmonary arteries, especially near its insertion near LPA and increased diastolic velocity >0.42 m/s across left pulmonary artery origin is associated with an hsPDA [21] (Figure 4f). The increased pulmonary blood results in increased pulmonary venous return, leading to ***dilated pulmonary veins*** and increased ***pulmonary vein Doppler flow***. The increased pulmonary venous return causes the LA to dilate. As the aortic valve annulus (Ao) is relatively fixed, the ***LA/Ao ratio*** is a surrogate for increased pulmonary venous return [21,22]. The mitral valve E/A ratio measures the inflow velocity during the early (E) diastolic phase of ventricular filling compared to the late atrial (A) contraction phase. Typically, the E/A ratio is >1, but in the presence of LA and LV volume overloading from the PDA shunt, this ratio is reversed, and the E/A ratio becomes <1. MV inflow (E-wave) slows down with increased LV volume and pressure, and there is an increased ‘A’ velocity from atrial contraction to push extra LA volume left after the initial diastolic phase (E-wave) (Figure 4c). Elevated LA pressure may cause early mitral valve opening or shorten ***isovolumic relaxation time (IVRT)***. The subsequent increase in LV volume can be quantified by measuring the ***LV end-diastolic dimension (LVEDD)***, adjusting to gestational age and body weight (z-score) [23]. Sometimes, the left heart dilation is noticeable enough to be visually observed as an ***LV/RV size discrepancy***. ***Mitral valve insufficiency*** may be seen in infants with hsPDA and a significantly dilated LV [23] (Figure 4b). Higher LV volume enhances ***LV cardiac output (LVO)*** and stroke volume, which can be calculated using ***velocity time integral (VTI)*** and Ao size [24]. If there is a significant intra-atrial or intra-ventricular shunt to alleviate volume overload in the left heart, it might result in reduced or delayed left heart dilation. Without an intracardiac shunt, an hsPDA may have a higher ***LVO to superior vena cava (SVC) flow ratio*** because the SVC is the surrogate for the return volume of preductal systemic blood flow [11,21]. 

Many echocardiographic parameters have been described [17,21]. The commonly used in clinical practice, which offer higher sensitivity and specificity, are: increased LPA diastolic velocity, an increased LA/Ao ratio, a qualitative assessment of left heart dilatation, and increased LVEDD [17,21] (Figure 4). To measure the LPA diastolic velocity, a pulsed wave Doppler is used to interrogate LPA blood flow in the parasternal short axis view. A mean and end-diastolic velocities of >0.42 m/s and >0.2 m/s, respectively, are highly suggestive of an hsPDA with a sensitivity and specificity of >90% [21] (Figure 4f). The LA/Ao ratio can be obtained from the parasternal long-axis view or the parasternal short-axis view using an M-mode cursor across the aortic valve level and left atrium. An LA/Ao ratio of >1.4 is suggestive of an hsPDA [22] (Figure 4d,e). LVEDD can also be measured from the parasternal long-axis view with an M-mode cursor at the tip of the MV leaflet or by placing the cursor perpendicular to the mid-LV cavity at the level of the papillary muscle in the parasternal short-axis view. Measured LVEDD values higher than the normative z-score adjusted to body weight and postnatal age suggest LV volume overload and a dilated left ventricle [23]. A rapid qualitative assessment on visualization of the heart chambers in apical 4-chamber view and the parasternal long axis view can be done—a significantly dilated LA/LV compared to the RA/RV is noted on “eyeballing,” but this remains subjective and may not be precisely accurate.

**Figure 5:**
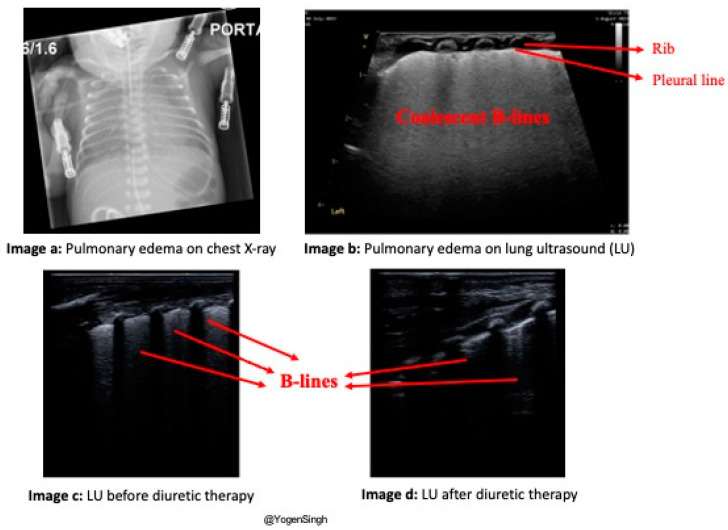
Assessment of pulmonary edema on chest X-ray and lung ultrasound (LU). (**a**) shows pulmonary edema with increased opacity on the chest X-ray. (**b**) shows severe pulmonary edema with a white-out appearance on LU. The white-out appearance results from coalescent B-lines, which are echogenic vertical lines below the pleural line, indicating interstitial pulmonary fluid. (**c**) shows severe pulmonary edema with multiple B-lines before the diuretic therapy. (**d**) shows fewer B-lines, suggesting decreased pulmonary edema after the diuretic therapy. *LU—lung ultrasound*.

A chest X-ray can be performed to assess the severity of pulmonary edema. A lung ultrasound can assess the severity of pulmonary edema, guide diuretic therapy, and monitor the response to interventions [25]. A lung ultrasound demonstrated increased echogenic vertical lines, known as B-lines, in the presence of pulmonary edema (Figure 5).

### 3.3. Echocardiographic Assessment of Systemic Hypoperfusion

**Figure 6:**
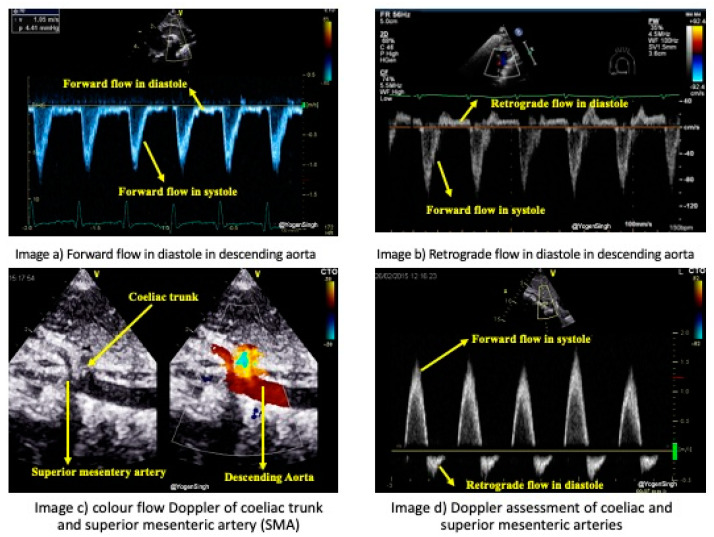
Doppler assessment of blood flow in the descending aorta post-ductally, in the coeliac trunk, and the superior mesenteric artery. (**a**) shows forward blood flow during systole and diastole in the descending aorta from the suprasternal arch view. (**b**) shows retrograde blood flow during diastole in the descending aorta, indicating ‘ductal steal’ in the presence of an hsPDA. (**c**) shows the coeliac and superior mesenteric arteries from the subcostal longitudinal color compare view, and (**d**) shows retrograde blood flow during diastole in the coeliac and superior mesenteric arteries, indicating ‘ductal steal’ similar to flow in the descending aorta.

Systemic hypoperfusion occurs in the presence of an hsPDA when blood diverts away from the systemic circulation below the ductus arteriosus level. Doppler evaluation can be used to assess the degree of systemic steal in which retrograde or absent blood flow is seen during the diastole in the post-ductal descending aorta, coeliac trunk, superior mesenteric artery, or renal artery [11,17,21,23,26,27]. Doppler flow patterns can be acquired in the descending aorta from the suprasternal view by distally positioning the pulsed wave Doppler sample gate to the ductal ampulla (Figure 6a,b). A Doppler assessment of the coeliac trunk or superior mesenteric artery is obtained from the subcostal longitudinal view—a pulsed wave Doppler sample gate is placed in the coeliac or superior mesenteric artery after optimizing the image so that the angle of insonation is minimal, <10–15 degrees is recommended. (Figure 6c,d) [17,26]. A Doppler assessment of the anterior or middle cerebral artery can be used to evaluate the hsPDA’s effect on brain perfusion, and a similar flow pattern is seen—absent or retrograde flow during diastole is observed in the presence of an hsPDA.

### 3.4. Echocardiographic Assessment of Myocardial Function

A hemodynamically significant PDA induces hyperdynamic cardiac status by increasing the LV preload from the PDA shunt volume, which can affect myocardial functions. Ventricular contractility can be assessed qualitatively on visualization, often called “eyeballing”, and assessment in multiple views (apical 4-chamber, parasternal long, and parasternal short axis views) is recommended to improve the accuracy. The increased cardiac functions can be evaluated objectively by using quantitative assessment tools such as fraction shortening (FS%), ejection fraction (EF%), or using more advanced functional echocardiographic methods, e.g., tissue Doppler imaging, the myocardial performance index, or speckle tracking and strain rate. Initially, systolic cardiac function typically remains preserved. However, persistent volume overloading of the LV may eventually impair its diastolic function, leading to a reversed mitral valve inflow E/A ratio on a Doppler assessment and, eventually, heart failure. 

## 4. A Practical Approach to Evaluate an hsPDA

The commonly used echocardiography parameters and indicative hsPDA values that can help clinicians assess the PDA and its hemodynamic significance have been summarized in Table 1. All echocardiographic parameters discussed above have limitations and should not be used to define an hsPDA in isolation. For instance, a significant inter-atrial shunt (across the patent foramen ovale or atrial septal defect) may offload the LA and reduce left atrial pressure and size; hence, using LA/Ao size to define an hsPDA may underestimate the degree of pulmonary overcirculation. Therefore, various comprehensive staging and scoring systems have been developed. Instead of relying on a single parameter, these proposed scoring systems combine different echocardiographic parameters to assist clinical decision-making [11,15,28]. One such system is the PDA Severity Scoring system proposed by El-khuffash et al. [28]. This scoring system calculates the PDA Severity Score using the following formula: [GA (in weeks) × 1.304 + PDA diameter (in mm) × 0.781 + LVO (ml/kg/min) × 0.008 + max PDA velocity (m/s) × −1.065 + LV a’ wave (cm/s) −0.470 + 41] [28]. A value of >5 out of 13 predicts chronic lung disease (CLD) or death associated with hsPDA with a sensitivity of 92%, specificity of 87%, positive predictive value of 92%, and negative predictive value of 92% [28]. Some staging and scoring systems share similar principles, requiring multiple complex echocardiographic measurements needing advanced imaging expertise and more time for evaluation, making them impractical for routine use in a busy clinical practice, and they are currently not being used by pediatric cardiologists [11,15,28]. Further research is necessary to develop a user-friendly PDA scoring system with validated sensitivity and specificity, which, more importantly, can be used by both neonatologists and pediatric cardiologists.

## 5. Clinical Decision-Making on Treating hsPDAs

A comprehensive evaluation of PDA and clinical decision-making to treat a hemodynamically significant PDA can be complex [27,30,31,32]. Echocardiographic parameters indicating the presence of an hsPDA may not always be clinically relevant, as they can be present in a healthy infant requiring minimal to no ventilatory support. On the other hand, an infant with similar echocardiographic parameters may have significant hemodynamic instability and other co-morbidities such as an increased need for respiratory support, oxygen needs, pulmonary hemorrhage, feed intolerance, renal impairment, etc. Furthermore, there is a high rate of spontaneous closures of PDAs, while pharmacological or procedural treatments are not risk-free [30]. Ultimately, managing an hsPDA requires individualized precision medicine after carefully considering various factors and individual patient circumstances. A detailed discussion on the management of PDA is out of the scope of this paper, but it has been discussed in the other manuscript of this series on PDA in preterm infants.

## 6. Conclusions

The ideal approach to managing a PDA in extremely premature infants is still under debate and currently lacks consensus. However, there is widespread agreement that a comprehensive echocardiographic evaluation to assess a PDA can lead to a more individualized precision medicine approach for choosing a patient-specific best treatment option. Before considering any medical or surgical PDA intervention, infants should undergo a detailed echocardiographic evaluation to rule out underlying CHD and assess the impact of the PDA shunt on pulmonary and systemic circulation. More studies using comprehensive echocardiographic evaluation criteria for intervention and advanced echocardiographic technologies, like tissue Doppler imaging and 3D echocardiography, may improve understanding and decision-making regarding the treatment of hsPDAs in the future.

## Figures and Tables

**Table 1:** Summary of commonly used echocardiographic parameters for PDA assessment and their values suggest an hsPDA in preterm infants (adapted from Singh Y et al. 2020) [17].

PDA Evaluation Criteria	Essential Echocardiographic Parameters for Assessment of PDA and Hemodynamic Evaluation (See Text for Details)	Less Commonly Used Echocardiographic Parameters (See Text for Details)
Ductal characteristics	PDA size (Small < 1.5 mm, Moderate 1.51–2 mm, Large > 2 mm) **AND** Flow direction (Left-to-right, right-to-left, or bi-directional) **AND** Doppler assessment for flow pattern across PDA (constricting or pulsatile pattern—maximum end-diastolic velocity to peak systolic velocity ratio measured to determine the type of flow pattern)	-
Assessment of pulmonary hyperperfusion	Volume overload of left heart on visual inspection **AND** LPA diastolic velocity (mean velocity > 0.42 m/s, end-diastolic velocity > 0.2 m/s) **OR** LA/Ao ratio * (mild < 1.4, moderate 1.5–1.8, severe > 1.8) **OR** LVEDD (z-score > +2 standard deviation) Document the presence or absence and magnitude of inter-atrial shunt	Mitral E/A ratio [17,29] Left ventricular cardiac output (LVO) [29] LVO to superior vena cava (SVC) flow ratio Pulmonary vein Doppler Isovolumic relaxation time (IVRT) [29] Mitral regurgitation [17]
Assessment of systemic hypoperfusion	Retrograde or absent blood flow during diastole in: - descending aorta **OR** - coeliac trunk, superior mesenteric, or renal artery **OR** - anterior or middle cerebral artery	
Assessment of myocardial function	Qualitative assessment of cardiac contractility on visual inspection Semi-quantitative and quantitative assessment of LV function by assessing fraction shortening (FS%) or ejection fraction (EF%)	Tissue Doppler Imaging Myocardial performance index using Tissue Doppler evaluation [29] Speckle tracking and strain rate

* A comprehensive echocardiographic assessment should be performed to rule out any underlying congenital heart defect (CHD) or pulmonary hypertension before any intervention. *Ao—aortic valve, LA—left atrium, LPA—left pulmonary artery, LV—left ventricle, LVEDD—left ventricular end-diastolic diameter, LVO—left ventricular output, SVC—superior vena cava, mm—millimeter, m/s—meter per second.*
